# Phylogenetic and molecular characterization of equine H3N8 influenza viruses from Greece (2003 and 2007): Evidence for reassortment between evolutionary lineages

**DOI:** 10.1186/1743-422X-8-350

**Published:** 2011-07-14

**Authors:** Maria Bountouri, Eirini Fragkiadaki, Vasileios Ntafis, Theo Kanellos, Eftychia Xylouri

**Affiliations:** 1 Department of Anatomy and Physiology of Farm Animals, Faculty of Animal Science and Aquaculture, Agricultural University of Athens, Greece; 2 Pfizer Animal Health, Biologicals Development, Ramsgate Road, Sandwich, CT13 9NJ, UK

## Abstract

**Background:**

For first time in Greece equine influenza virus infection was confirmed, by isolation and molecular analysis, as the cause of clinical respiratory disease among unvaccinated horses during 2003 and 2007 outbreaks.

**Methods:**

Equine influenza virus (EIV) H3N8 was isolated in MDCK cells from 30 nasal swabs from horses with acute respiratory disease, which were tested positive by Directigen Flu A. Isolation was confirmed by haemagglutination assay and RT-PCR assay of the M, HA and NA gene.

**Results:**

HA sequences of the Greek isolates appeared to be more closely related to viruses isolated in early 1990s in Europe. These results suggested that viruses with fewer changes than those on the main evolutionary lineage may continue to circulate. On the other hand, analysis of deduced NA amino acid sequences were more closely related to viruses isolated in outbreaks in Europe and Asia during 2003-2007. Phylogenetic analysis characterized the Greek isolates as a member of the Eurasian lineage by the haemagglutinin (HA) protein alignment, but appeared to be a member of the Florida sublineage clade 2 by the neuraminidase (NA) protein sequence suggesting that reassortment might be a possible explanation.

**Conclusion:**

Our findings suggest that the Greek strains represent an example of "frozen evolution" and probably reassortment between genetically distinct co-circulated strains. Therefore expanding current equine influenza surveillance efforts is a necessity.

## 1 Background

Equine influenza viruses (EIVs) are the etiologic agents of severe epidemic respiratory disease in horses. Antigenically they are classified as influenza type A viruses belonging to the family *Orthomyxoviridae *containing 8 single stranded RNA molecules of negative polarity. Influenza infections are frequently followed by secondary bacterial disease, with serious and sometimes life-threatening consequences for the horses. The rapidly spreading signs include high pyrexia, dyspnoea, coughing, myalgia, anorexia and swelling of regional lymph nodes [1].

The first EIV to be isolated was influenza A/equine/Prague/56 (H7N7) in 1956 [2]. However, the H7N7 subtype has not been isolated from horses for over 20 years and is presumed not to circulate at present [3]. A second subtype of the virus, A/equine/Miami/63 (H3N8), was isolated during a major epizootic of respiratory disease in the U.S.A in 1963 [4]. The H3N8 subtype is responsible for widespread outbreaks in vaccinated and unvaccinated horses. Neither of the subtypes cross-reacts immunologically and therefore natural infection or vaccination with one subtype will not protect against infection by the other [5].

In 1996, Daly and co-workers, demonstrated that in 1989 a divergent evolution of American and European isolates had occurred resulting in two genetically and antigenically distinct lineages. The Eurasian, included stains circulating mostly in the European continent and the prototypes are A/equine/Newmarket/2/93 and A/equine/Suffolk/89, and the American lineage included stains detected prevalently in the American continent and the prototype is A/eq/Newmarket/1/93, while the oldest H3N8 strains, circulating in the '70s and '80s are now apparently extinct [6]. The American lineage was further distinguished in Argentina, Kentucky and Florida sub-lineage, and the last has been divided in clade 1 and 2 [7]. Clade 1 includes the A/eq/Wisconsin/03-like viruses while clade 2 is represented by the A/eq/Newmarket/5/03-like viruses [8]. At present, viruses of the two lineages co-circulate in horse populations, therefore it was suggested that vaccines contain strains representative of those currently circulating in the field [9]. During 2003-2007 widespread outbreaks of EIV have been reported not only in many countries of Europe and in USA, but also in regions that rarely report EI outbreaks [10-12]. Even, Australia, a country previously free of equine influenza, suffered an outbreak in 2007 [13].

In addition to the linear evolution of HA, the segmented nature of the influenza virus genome allows reassortment to take place resulting in rapid virus evolution [14]. Reassortment is significant if it occurs between distinct co-circulating viral strains. Nucleotide analysis of H3N8 viruses have shown reassortment of RNA segments encoding NP [15,16], PB2 [17] and PA [18] between the equine H7N7 and H3N8 subtypes and of segments of HA and NS between distinct strains of H3N8 strains [11].

Here, we describe for first time the successful isolation and characterization of EIV from horses in Greece from two outbreaks (in 2003 and 2007). Moreover our report includes information on sequencing analysis and phylogenetic relationship of HA and NA proteins of the Greek isolates.

## 2 Materials and methods

### 2.1 Outbreak description - Sampling

In June 2003 and in May 2007, an acute respiratory disease was reported in the same stud farm in Attiki, Greece. 20 (in June 2003) and 10 horses (in May 2007) were affected and had pyrexia, nasal discharge, anorexia, dyspnoea, cough, myalgia and general depression. Signs lasted 5-10 days. The age of the infected animals varied from 6 months to 2 years. Symptomatic treatment with antibiotics, antipyretics and nonsteroidal anti-inflammatory drugs (NSAIDs) had limited results. All infected horses were unvaccinated against equine influenza virus. Nasal swabs were collected from the 30 affected animals, between the 3^rd ^and 4^th ^day after the onset of clinical signs. All nasal swabs collected were Directigen Flu A positive when tested on the spot at the time of sampling. No serum samples were submitted from the affected animals. During the 2007 outbreak there was none of the 2003-outbreak affected animals still in the farm.

### 2.2 Virus isolation

All samples were placed in virus transport medium and brought on ice within one hour from the time of sampling to the laboratory. Nasal secretions were centrifuged and stored at -80°C. After thawing, 100 μl of each filtered swab was used for inoculation of 5 × 10^4 ^MDCK cells/per well in 6-well plates. After 48-72 h incubation at 37°C (under 5% v/v CO_2 _humidified conditions) a blind passage of each passage was performed. In total, three successive passages per sample were conducted and cell cultures were checked daily for the presence of cytopathic effect (CPE).

Cell viability was determined at 72 h at each passage using a Trypan blue (0.4% w/v) exclusion assay. Cell viability was counted using a hemocytometer, and expressed as the percentage of live cells in the infected cell cultures in comparison to the control (uninfected) cell culture (no of live cells in infected cell culture/live cells in control cell culture × 100%).

Quantification assays to determine the TCID_50 _were carried out at the third passage of the isolated samples [19].

### 2.3 Antigenic characterisation of equine influenza virus isolates

Haemagglutination inhibition was carried out as described previously [5] against 3 post infection ferret antisera treated with periodates and heated to remove non specific haemagglutination inhibitors. The ferret antisera used were against Newmarket/1/93 (H3N8), Newmarket/2/93 (H3N8) and Newmarket/77 (H7N7). The strain Newmarket/1/93 was used as positive control virus in all assays

### 2.4 RNA extraction, RT-PCR, sequencing and phylogenetic analysis

Viral RNA was isolated from nasal swabs and cell culture supernatant using the QIAampViral RNA mini kit (Qiagen) according to the manufacturer's instructions. Genes of interest were amplified using the SuperScript One-Step RT-PCR Platinum Taq (Invitrogen S.R.L.) with 10 μl of template RNA using the gene specific primers M52C and M253R described by Fouchier et al, 2000 for Matrix gene [20], primers H3HA1/RT and H3HA1/2 described by Newton et al. 2006 for HA1 [21] and primers NA25 and NA703 described by Ito et al., 2008 for NA gene (position 25-703) [10] at a final concentration of 0.2 μM. The cycling conditions used were the same as described previously [10,20,21]. Although, previous reports have used GeneAmp PCR kit (Applied Biosystems) for the nested PCR, during this study another second (nested) PCR was carried out for HA gene using HotStartTaq DNA Polymerase (Qiagen) kit. 2.5 μl of the product of the first RT-PCR step were amplified in a final volume of 50 μl containing, 0,2 μM each of sense (H3HA1/3) and antisense (H3HA1/4) primers. The mixture was heated at 95°C for 5 min, followed by 35 cycles of 94°C for 30s, 50°C for 1 min and 72°C for 1 min, and finally 7 min at 72°C.

PCR products were analysed on a 1% agarose gel stained with ethidium bromide and purified using the QIAquick PCR Purification Kit (Qiagen) according to the manufacturer's instructions. PCR products of each gene specific PCR assay were sequenced using DyeDeoxy Terminator Cycle Sequencing on an AB37710xl Genetic Analyser (Applied Biosystems). Sequences were assembled using Bioedit software package version 3.1 [22] and compared to cognate sequences in the genetic databases using BLAST web-based program http://www.ncbi.nlm.nih.gov/BLAST. Nucleotides as well as deduced amino acid sequences of HA and NA genes were aligned by using Clustal W method and unrooted phylogenetic trees were generated using the MEGA software version 4.0 [23] Phylogenetic trees were constructed using the NJ method and the aminoacid sequences. The statistical analysis of the trees was assessed by bootstrap resampling (1000 data sets) of the multiple alignments.

## 3 Results

A total of 30 nasal swabs were received from the two outbreaks, which were tested positive with immunoassay Directigen Flu A; 16 of these were isolated in MDCK cells, 15 of these showed haemagglutination activity and 18 clinical specimens were positive in RT-PCR reaction of the M, HA and NA gene.

### 3.1 Isolation and antigenic characterisation

Influenza A virus was isolated from nasal swabs collected from 11 out of 20 and 5 out of 10 affected animals in the 2003 and 2007 outbreak, respectively.

**Figure 1:**
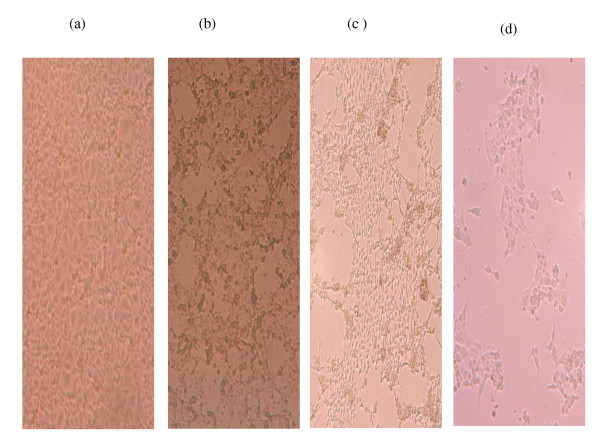
**CPE of equine influenza virus: MDCK cells incubated in the absence [control (a)] and presence of 100 μl swab extract for 24 h (b), 48 h(c) and 72 h (d)**. (Invertoscope, 10× magnification).

**Figure 2:**
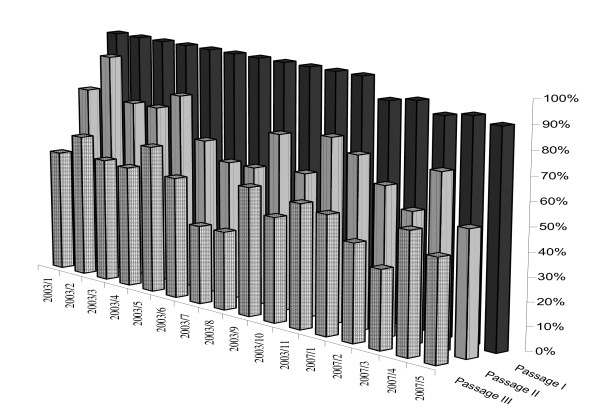
**Viability of MDCK cell cultures 72 h post infection at each passage expressed as a percentage of live cells in comparison to the control cell culture**.

MDCK cells showed cytopathic effects (CPE). There was no or slight apoptosis after the first passage. Two successive passages followed and apoptosis was observed at 48 h and 72 h post -infection. Figure 1 shows CPE effects of infected MDCK cells and Figure 2 shows the results of the trypan blue assay.

Although, influenza virus was propagated in the MDCK cell line, the strains showed low haemagglutination (HA) titres following three passages. The results of the virus isolation, of titration (TCID_50_) and of haemmaglutination of the isolates are summarised in Table 1. The haemagglutination titre increased by each passage in most of the samples, but in some specimens there was no HA titre increase between the passages. Specimen 2003/9, was the only which showed no haemagglutination, although CPE was observed and PCR assay (on the 3^rd ^passage) was positive. HI titres were obtained only with Newmarket/2/93.

**Table 1:** Results of Virus Isolation confirmed by RT-PCR assay for the M gene (at the 3rd passage), hemagglutination titer, and TCID50 of the isolates at the 3rd passage

	HA titer				
			
**Horse no**.	Passage I	Passage II	Passage III	VI*	TCID 50/ml
**2003/1**	0	4	4	+	10 ^1,25^
**2003/2**	0	2	4	+	10 ^0,75^
**2003/3**	2	4	32	+	10 ^1,75^
**2003/4**	0	4	8	+	10 ^1,75^
**2003/5**	2	4	8	+	10 ^0,5^
**2003/6**	2	4	8	+	10 ^1,25^
**2003/7**	0	2	4	+	10 ^2,25^
**2003/8**	0	2	4	+	10 ^2,25^
**2003/9**	0	0	0	+	10 ^1,5^
**2003/10**	0	8	32	+	10 ^2,75^
**2003/11**	0	0	4	+	10 ^1,75^
**2007/1**	2	4	8	+	10 ^1,5^
**2007/2**	4	8	16?	+	10 ^2^
**2007/3**	0	4	32	+	10 ^2,25^
**2007/4**	4	8	32	+	10 ^1,75^
**2007/5**	4	16	32	+	10 ^2,5^

* Confirmed by RT-PCR for the M gene at the 3^rd ^passage

### 3.2 Molecular Analysis

2003 samples: equine influenza virus was detected by RT-PCR assay (a band of 244 bp) in 11 out of 20 swab extracts and from all 11 cell culture extracts after 3 passages in MDCK cells.

2007 samples: equine influenza virus was detected by RT-PCR assay in 7 out of 10 swab extracts and from all 5 cell extracts after 3 passages in MDCK cells.

Moreover the RT-PCR for the detection of HA3 and NA8 equine influenza A subtype amplified a band of 884 bp and a band of 678 bp, respectively in the same samples from both outbreaks.

During our experiments incompatibility was found between SuperScript One-Step RT-PCR Platinum Taq (Invitrogen S.R.L.) (used at the RT-PCR for HA gene) and GeneAmp PCR kit (Applied Biosystems) (used at the nested PCR for HA gene). There were no results by using GeneAmp PCR kit (Applied Biosystems), not even for the positive control.

The detailed analysis of the sequences of HA and NA genes obtained of both outbreaks were deposited in GenBank (accession nos. NCBI:HM164058, NCBI:HM164059, NCBI:FJ605181, NCBI:FJ605182 and NCBI:HM164056, NCBI:HM164057, NCBI:HM164055, NCBI:HM164054 respectively) and revealed 100% similarity between them. BLAST analysis of the partial sequence of HA and NA genes confirmed the H3N8 subtype of the isolate. Comparison of nucleotide sequences of the Greek isolates with published equine influenza virus sequences using BLAST is showed in details in Table 2.

**Table 2:** Comparison of nucleotide sequences of Greek virus with published equine influenza virus sequences using BLAST web-based program

	Most similar strains	Similarity %
HA gene	A/eq/Newmarket/2/93/(H3N8) A/equine/Italy/1199/1992(H3N8) A/equine/Berlin/1/91(H3N8) A/equine/Roma/5/1991(H3N8) A/equine/Switzerland/173/1993(H3N8) A/swine/Anhui/01/2006(H3N8) A/equine/Switzerland/P112/2007(H3N8) A/equine/Berlin/14/2002 (H3N8) A/equine/Leicestershire/1/00(H3N8) A/equine/Lincolnshire/1/2002(H3N8)	100% 99% 99% 99% 99% 98% 98% 98% 98% 98%
NA gene	A/equine/Gansu/7/2008(H3N8) A/equine/Spain/1/07 H3N8 A/equine/Spain/1/09 H3N8 A/equine/Huabei/1/2007(H3N8) A/equine/Newmarket/5/2003(H3N8) A/equine/California/8560/2002(H3N8) A/equine/Kentucky/5/2002(H3N8) A/equine/Newmarket/1/1993(H3N8)	99% 99% 99% 99% 99% 99% 99% 97%

HA gene alignment analysis of the Greek isolates showed no nucleotide substitution among them and in comparison with A/equine/Newmarket/2/93 strain. When the amino acid sequences of our isolates were aligned with a virus isolated in Switzerland in 2007 six amino acid substitutions (positions 43D-V, 100G-R, 123G-E, 209M-T, 238L-P, 265I-V) were seen.

To define amino acid differences within NA, multiple alignment of the deduced amino acid sequences of Greek strains and representative H3N8 strains including vaccine strains was performed. Although Gansu/08, was defined as most similar strain to ours using BLAST, they had two nucleotide point mutations which lead to two amino acid differences, 209I → V and 229C → R. Multiple alignment revealed three nucleotide mutations and three animo acid differences between our strains and Huabei/07 (209I → V, 218E → A, 229C → R). There were observed 6 amino acid substitutions between Newmarket/5/03 and Greek isolate in positions 9, 12, 40, 66, 191, 229 and Newmarket/5/03 versus Gansu/08 in positions 9, 12, 40, 66, 191, 209. Greek isolates shared 99% similarity with isolates from Spain (A/equine/Spain/1/2007 and A/equine/Spain/1/2009) when nucleotide sequences were compared and three (R150Q, T180A, R229C) and two (N29P, R229C) aminoacid substitutions respectively

**Figure 3:**
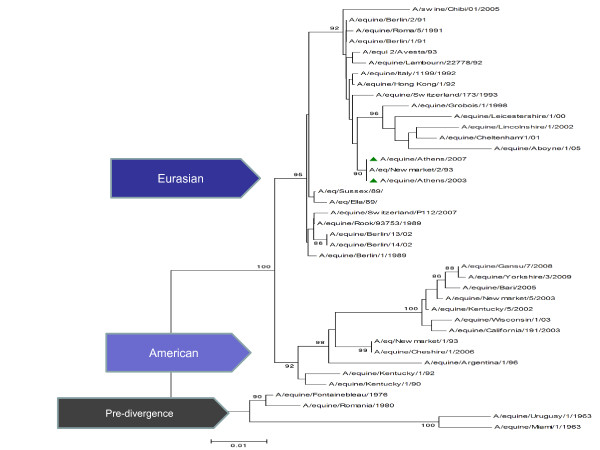
**Evolutionary relationships of H3 protein**: The evolutionary history was inferred using the NJ method. The percentage of replicate trees in which the associated taxa clustered together in the bootstrap test is shown next to the branches. The tree is drawn to scale, with branch lengths in the same units as those of the evolutionary distances used to infer the phylogenetic tree. The evolutionary distances were computed using the Kimura 2-parameter method and are in the units of the number of base substitutions per site. Phylogenetic analyses were conducted in MEGA4.

**Figure 4:**
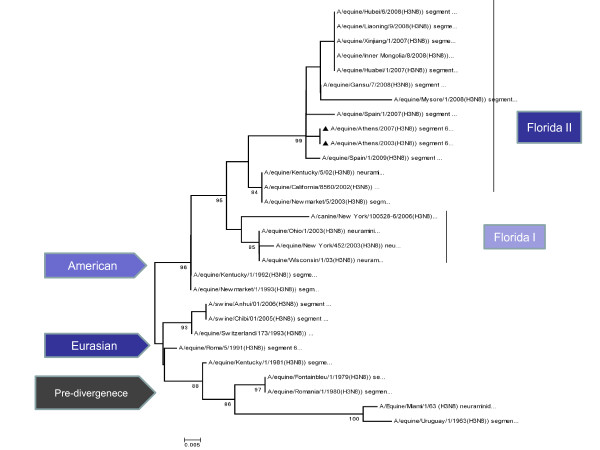
**Evolutionary relationships of N8 protein**: The evolutionary history was inferred using the NJ method. The percentage of replicate trees in which the associated taxa clustered together in the bootstrap test is shown next to the branches. The tree is drawn to scale, with branch lengths in the same units as those of the evolutionary distances used to infer the phylogenetic tree. The evolutionary distances were computed using the Kimura 2-parameter method and are in the units of the number of base substitutions per site. Phylogenetic analyses were conducted in MEGA4.

Phylogenetic analysis of the HA protein of our isolates clustered the virus among the Eurasian lineages, but by the NA protein appeared to be a member of the American lineages and in particular the Florida sub-lineage clade II. Phylogenetic trees constructed with NJ method using the HA and NA amino acid sequences are shown in Figure 3 and Figure 4. The topology of the maximum-likehood tree for both HA and NA sequences showed four clades corresponding to the Pre-diverge, American, Eurasian and Florida lineage viruses. The Greek isolates were well supported within the Eurasian clade, as seen for the HA protein with bootstrap value 90% and 99% within the Florida sublineage clade II for the NA protein.

## 4 Discussion

The objective of our study was to isolate and characterize the influenza virus causing respiratory disease outbreaks in unvaccinated horses, which were Directigen Flu A positive, in Athens during 2003 and 2007 outbreaks. The results from all assays revealed that the causative agent of the outbreaks was an equine H3N8 influenza virus. This is the first report of molecular and phylogenetic analysis of the virus in Greece.

Equine influenza virus was isolated in MDCK continuous cell line from 16 samples (out of 18 RT-PCR positive samples) from both outbreaks. This is a comparatively high percentage (89%) as Morley et al. (1995) [24] reported 7% influenza virus isolations from nasal swab extracts obtained from horses with acute respiratory disease during influenza epidemics. According to our study, isolation in MDCK proved to be a good assay as it showed many positive results. Moreover, a comparative sequence analysis of the HA gene was conducting between the clinical specimens and cells culture extracts which resulted in a homogenous preparation with respect to the HA gene. This was not in agreement with previous studies which support the use of embryonated eggs duo to lack of selection of changes in the HA compared to their propagation in cells [25,26]. On the other hand, the propagation of the virus in cell lines and not in embryonated eggs may be responsible for the low haemagglutination titres.

The Greek isolates were classified as members of the Eurasian lineage analysing the HA sequences and Florida sub-lineage member viruses seen with the NA sequence. Reassortment between a Eurasian and a Florida sub lineage clade II strain might be responsible for this. Our findings are with compliance with previous reports of reassortment between genes of district lineages. Bryant et al., referred that A/equine/Lincolnshire.1/06 obtained an European-like NS gene and a Florida Clade II- like HA gene [11]. It was suggested that circulating Florida sub-lineage virus strains may be able to acquire gene segments from a simultaneously circulating Eurasian strain, potentially resulting in changes in pathogenicity [11]. Differences in pathogenicity have been observed between Eurasian and American lineage viruses in experimental infection of ponies [27,28].

One possible explanation of the Eurasian lineage-like HA gene is that the virus isolated from the two outbreaks in Greece is an example of "frozen evolution". Endo et al (1992) [29] was the first who described this phenomenon, which refers to viruses that appear to date from earlier isolates. A/eq/Switzerland/07, the latest isolate being characterized as a Eurasian lineage virus is an example of "frozen evolution". A/equine/Cheshire/1/06, which showed 100% similarity to the prototype strain A/equine/Newmarket/1/93, was another evidence of frozen evolution as it was characterised as a member of the American lineage [11]. This may result from a reduced amount of antigenic drift compared to the majority of circulating EIVs.

Another possible explanation for isolation of viruses that are similar to virus reference strains, is laboratory contamination or vaccine origin as suggested previously [30]. Laboratory contamination was unlikely in our case, as none of the strains to which the A/equine/Athens viruses are most closely related are handled in our laboratory. In addition to this, the two set of isolates from 2003 and 2007, were inoculated in MDCK cells and tested by RT-PCR assay with two years difference. It is worthwhile to refer that all the horses in our study were unvaccinated as in most of previous reports of frozen evolution [11,29,31-33] isolates were from unvaccinated animals or animals with uncertain vaccination history. One possible explanation of the small number of isolates belonging to this lineage may be that these viruses are circulating among unvaccinated animals or the Eurasian lineage may be dying out. However, Tu et al., demonstrated that an H3N8 equine influenza strain member of the European strain transmitted to pigs in China in 2005 and 2006 [34]. The sequencing analysis of the eight gene segments of the swine isolates revealed that they were more closely related to European equine influenza viruses from the early 1990s. This is evidence that the horse may not be the dead-end host. Thus, members of this lineage continue to circulate among equine populations or periodically reintroduced into horse populations from an unknown source [34].

The precedent for co-circulation of multiple lineages of EIVs over a number of years without one replacing the other, reinforce the possibility of reassortment reported here. Co-circulation of EIVs led to concerns that in future viruses that show evolution stasis may re-emerge from unvaccinated populatios and threaten vaccinated populations [33].

In order to clarify whether this was only a sporadic phenomenon, and to determine the origin of the reassorted strains further sequencing analysis of the genome H3N8 viruses needs to be performed.

## Conclusion

In conclusion, the present study is the first report of the successful isolation and genetic characterization of an H3N8 equine influenza virus that were causing respiratory disease in un-vaccinated horses in Greece. Strains isolated from both outbreaks in 2003 and 2007 were more closely related to older European lineage H3N8 subtype strains according to their HA sequences, indicating that older viruses may continue to circulate perhaps in unvaccinated horses. However, Greek strains appeared to be members of the Florida sublineage clade II by the NA gene sequencing. One possible explanation would be that the strains were derived by reassortment of co-circulated strains. Increased international movement of horses for breeding and competition purposes constitutes an important factor in the spread of equine pathogens throughout the world [12]. The circulation, spread and reassortment of genetically distinct strains is therefore not surprising. Further study is needed to determine the circulation dynamics of H3N8 influenza virus.

## Competing interests

The authors declare that they have no competing interests.

## Authors' contributions

MB (AB), carried out all the experiments, including designing the experiments, analysis and interpretation of data and drafting the manuscript. EF helped in acquisition of samples and revising the manuscript. VN participated in the sequence alignment (MT) and helped revising the manuscript. TK (ES) participated in the design of the study and EX. (FG) conceived of the study, and participated in its design and coordination. All authors read and approved the final manuscript. All authors have read and approved the final manuscript.
